# Preventing the development of depression at work: a systematic review and meta-analysis of universal interventions in the workplace

**DOI:** 10.1186/1741-7015-12-74

**Published:** 2014-05-09

**Authors:** Leona Tan, Min-Jung Wang, Matthew Modini, Sadhbh Joyce, Arnstein Mykletun, Helen Christensen, Samuel B Harvey

**Affiliations:** 1 University of New South Wales, School of Psychiatry, Black Dog Institute, Hospital Road, Randwick NSW 2031, Australia; 2 Norwegian Institute of Public Health, Bergen, Norway; 3 Black Dog Institute, Hospital Road, Randwick NSW 2031, Australia; 4 St George Hospital, Gray Street, Kogarah NSW 2217, Australia

**Keywords:** Depression, Prevention, Workplace, Occupational health, Occupational stress, Mental disorder, Resilience

## Abstract

**Background:**

Depression is a major public health problem among working-age adults. The workplace is potentially an important location for interventions aimed at preventing the development of depression, but to date, the mental health impact of universal interventions in the workplace has been unclear.

**Method:**

A systematic search was conducted in relevant databases to identify randomized controlled trials of workplace interventions aimed at universal prevention of depression. The quality of studies was assessed using the Downs and Black checklist. A meta-analysis was performed using results from studies of adequate methodological quality, with pooled effect size estimates obtained from a random effects model.

**Results:**

Nine workplace-based randomized controlled trials (RCT) were identified. The majority of the included studies utilized cognitive behavioral therapy (CBT) techniques. The overall standardized mean difference (SMD) between the intervention and control groups was 0.16 (95% confidence interval (CI): 0.07, 0.24, *P* = 0.0002), indicating a small positive effect. A separate analysis using only CBT-based interventions yielded a significant SMD of 0.12 (95% CI: 0.02, 0.22, *P* = 0.01).

**Conclusions:**

There is good quality evidence that universally delivered workplace mental health interventions can reduce the level of depression symptoms among workers. There is more evidence for the effectiveness of CBT-based programs than other interventions. Evidence-based workplace interventions should be a key component of efforts to prevent the development of depression among adults.

## Background

Organizations are increasingly recognizing their obligation to employee health as marked by the rise in workplace health initiatives, particularly over the last two decades [1-3]. Despite mental disorders being the leading cause of sickness absence and work incapacity in most developed countries [4,5], mental health has remained relatively ignored in the majority of workplace health programs. With depression predicted to be the leading cause of work disability by 2020 [6], there is a growing need for evidence-based workplace mental health interventions. To date, most work-based responses to mental health problems have been reactive, with interventions only being considered once a worker is symptomatic and often on sick leave [7]. However, recent evidence suggests that many mental health problems may be prevented [8], raising the prospect that workplaces might proactively prevent the onset of mental health problems. Despite the appeal of such strategies, to date there has been very little consensus on whether such preventative programs are effective in the workplace [9].

Workplaces have been suggested as an ideal site for prevention programs for a number of reasons [9]. First, with 60% of the world’s population engaged in some form of employment and 60% of their waking hours spent at the workplace, there is potential to reach a substantial number of people in a reliable and predictable manner [10]. Second, an adverse psychosocial work environment is established as a risk factor for mental disorder [11], meaning work-based interventions can be multi-modal in simultaneously reducing known risk factors while enhancing individual coping skills and resilience. Third, if found to be effective the cost of mental health interventions based in the workplace could be shared by both the private and health sectors. A recent review suggested that interventions focused on the prevention or treatment of mental health problems were likely to produce a favorable financial return on the investment [12].

Prevention programs can be directed at an entire population (universal prevention), only those at high risk (selective prevention), or only those with emerging symptoms (indicated prevention) [8]. Although the relative effectiveness of the different types of prevention as they relate to mental health remains unclear [13], there are theoretical and practical reasons that universal interventions may be most appropriate for the workplace. From a public health perspective, universal interventions are attractive not only because they can reach more working adults, but also because they can reach selected and indicated groups without the need for screening, which has been found to be a costly exercise [14,15]. Targeting an entire population also reaches individuals who might not want to seek treatment or disclose symptoms for fear of stigmatization and the perceived negative effects on employment [16]. Such fears may be particularly relevant in a workplace situation, where previous research has found evidence that prejudiced attitudes by employers towards individuals with depressive symptoms are common [17].

In settings outside of the workplace, preventive interventions using a variety of cognitive behavioral and psychotherapy techniques have been found to effectively reduce the incidence of mental disorders [13]. Only one review, which focused on literature published between 1997 and 2007, has specifically examined mental health interventions in the workplace. A small but positive effect on reducing symptoms of depression and anxiety was found, but the methodology of the review was limited by the inclusion of studies other than randomized controlled trials [18]. In the six years since this review, a number of new randomized controlled studies have been published. As a result, it is now timely to conduct a systematic review and meta-analysis of the evidence for work-based universal prevention of depressive illness.

## Methods

### Search strategy

A comprehensive literature search was conducted using the electronic databases MEDLINE, PsycINFO and EMBASE for relevant articles published from 1980 to January 2013. The search strategy was limited to these years since the first prevention randomized controlled trials for depression were conducted around 1980 [19,20]. A combination of keywords relating to the workplace, depression, interventions and randomized controlled trials were used. The search strategies created for all three databases are displayed in Table 1. To increase coverage, an additional search using the Cochrane Central Register of Controlled Trials (CENTRAL) was conducted using a combination of “mental health” and “work” search terms. The reference lists of all included studies from the above strategy were also scrutinized to identify any relevant publications that had not been considered.

**Table 1:** Search strategy terms

**Database**	**Workplace AND**	**Intervention AND**	**Outcomes AND**	**Study design**
**Medline**	employment.ti.	occupational intervention*.tw.	mental health.ti.	RCT.tw.
	job.ti.	occupational therap*.tw.	mental illness.ti.	randomized controlled trial.tw.
	work*.ti.	stress management.tw.	mental disorder*.ti.	random allocation.tw.
	worker*.ti.	stress inoculation training.tw.	psychiatric.ti.	random assignment.tw.
		resilience.tw.	depress*.tw.	exp randomized controlled trial/
	occupational health.tw.	resilience training.tw.		
	workplace.tw.	prevent*.tw.	mood disorder*.tw.	controlled clinical trial/
	work place.tw.	universal prevention.tw.		clinical trial/
	business*.tw.	primary prevention.tw.	exp depressive disorders/	random allocation/
		secondary prevention.tw.		
	exp industrial psychology/	self efficacy.tw.	affective symptoms.sh.	
	exp employment/		depression.sh.	
	exp Professional Corporations/	exp resilience, psychological/	mental disorders.sh.	
		exp primary prevention/	mental health.sh.	
	occupational health.sh.	exp self efficacy/		
	occupational exposure.sh.	exp secondary prevention/		
	occupational health services.sh.	exp Health Promotion/		
	occupational medicine.sh.			
	manage*.sh.			
**psycINFO**	employment.ti.	occupational intervention*.tw.	mental health.ti.	RCT.tw.
	job.ti.	occupational therap*.tw.	mental illness.ti.	randomized controlled trial.tw. random
	work*.ti.	stress management.tw.	mental disorder*.ti.	allocation.tw.
	worker*.ti.	stress inoculation training.tw.	psychiatric.ti.	random assignment.tw.
		resilience.tw.		
	occupational health.tw.	resilience training.tw.	depress*.tw.	treatment effectiveness evaluation/
	workplace.tw.	primary prevention.tw.	mood disorder*.tw.	exp Experimental Design/
	work place.tw.	secondary prevention.tw.		exp mental health program evaluation/
	business*.tw.	universal prevention.tw.	exp affective disorders/	
		prevent*.tw.	exp major depression/	
	exp occupational stress/	self efficacy.tw.	exp mental disorders/	
	exp personnel/		exp "Depression (Emotion)"/	
	exp working conditions/	exp Stress Management/		
	exp industrial psychology/	exp exposure therapy/	mental health.sh	
	exp Business Organizations/	exp prevention/		
	exp Management/	exp "resilience (psychological)"/		
		exp Self Efficacy/		
	occupational health.sh.	exp primary mental health prevention/		
	occupational safety.sh.	exp Health Promotion/		
	occupational stress.sh.			
	occupational neurosis.sh.	occupational stress.sh.		
	organizational behavior.sh.	occupational therapy.sh.		
	work related illnesses.sh.			
**Embase**	employment.ti.	stress inoculation training.tw.	mental health.ti.	RCT.tw.
	job.ti.	stress management.tw.	mental illness.ti.	randomized controlled trial.tw.
	work*.ti.	resilience.tw.	mental disorder*.ti.	random allocation.tw.
	worker*.ti.	resilience training.tw.	psychiatric.ti.	random assignment.tw.
		prevent*.tw.		
	occupational health.tw.	self efficacy.tw.	depress*.tw.	exp randomized controlled trial/
	work place.tw.	primary prevention.tw.	mood disorder*.tw.	exp controlled clinical trial/
	workplace.tw.	secondary prevention.tw.		
	business*.tw.	universal prevention.tw.	exp major depression/	exp randomization/
		occupational intervention*.tw.		
	exp management/	occupational therap*.tw.	exp mental health/	
			exp emotional disorder/	
	occupational exposure.sh.	exp stress management/		
	occupational health.sh.	exp primary prevention/	mood disorder.sh.	
	occupational psychology.sh.	exp health promotion/		
	occupational safety.sh.	exp secondary prevention/		
	work.sh.			
	workplace.sh.			

*, Retrieves all possible suffix variations of the root word indicated.

### Inclusion criteria

This review sought to identify all randomized controlled trials (RCTs) concerning workplace interventions that reported outcomes on a standardized mental health measure of depression. In order to be included in this review, the interventions needed to be aimed at universal prevention of depression within an entire workforce population. Studies had to compare at least two different randomly allocated intervention groups with at least one being a control or wait-list group. Participants of the studies had to be working-age adults (18 to 65 years) that belonged to a workgroup.

True preventive intervention studies require a standardized diagnostic tool at baseline to exclude the presence of disorder and to examine incidence at follow-up. However, as noted above, in a workplace situation it is often more practical to deliver prevention programs to an entire unscreened population, a strategy termed universal prevention. Given the difficulty of demonstrating true prevention in large clinical trials, studies of universal prevention without a baseline diagnostic assessment, testing universal symptom reduction in the workplace were also included in this review [21].

The majority of studies examining workplace mental health interventions utilize self-report scales of depressive symptomatology and as such, examine the reduction of depressive symptoms rather than prevention of diagnosed depression. In order to reduce this potential limitation, only studies utilizing established and validated measures of depression symptoms were included in this review. We will use the term “depression” to refer to high symptom loads as measured by a validated symptom scale. In order to ensure any effects were relatively persistent, studies had to include a follow-up of at least four weeks.

### Exclusion criteria

Articles excluded from the review were those that considered volunteer work, unemployed participants, focused on selected or indicated prevention, examined non-mental health outcomes and non-English publications.

### Quality assessment

The quality of the identified randomized controlled trials was assessed using the Downs and Black checklist [22]. This scale was identified as the most appropriate for the present review as it was specifically developed for the domain of public health. The Downs and Black checklist demonstrates strong criterion validity (r = 0.90) [23], good inter-rater reliability (r = 0.75) and has previously been used in a similar Cochrane Collaboration review [24]. The 27-item checklist is comprised of five subscales that measured reporting, external validity, internal validity (two subscales on bias and confounding) and power. As with previous studies [25,26], the tool was modified slightly for purposes of this review in that the scoring for question 27 on power was simplified to either zero or one-point based on whether or not there was sufficient power in the study to detect a clinically significant effect. Thus, studies reporting power of less than 0.80 with alpha at 0.05 obtained a zero score. The maximum score for the modified checklist was 28 with all individual items rated as either yes (= 1) or no/unable to determine (= 0), with the exception of item 5, “Are the distributions of principals confounders in each group of subjects to be compared clearly described?” in which responses were rated as yes (= 2), partially (= 1) and no (= 0). The ranges of scores were grouped into four categories: Excellent (26 to 28), good (20 to 25), fair (15 to 19) and poor (14 and less). Studies with an overall “poor” quality assessment were excluded from the final review.

### Data extraction

A data extraction sheet was designed to record the data. The variables extracted included sample characteristics, research design (individual or clustered RCT), implementation characteristics (intervention type) and outcome indicators. All data required for the calculation of effect sizes were entered into the R v.2.15.2 statistical programming language [27].

### Contact with authors

Where there were missing data or additional information was required for effect size calculations, study authors were contacted. The contact details of the authors were obtained through the correspondence addresses on the study reports; website searches were also performed to ensure that the contact emails were still in use and valid. Authors were all contacted by email, and all non-responders were sent a follow-up email one to two weeks later.

### Data synthesis/statistical analysis

Our main analysis was conducted using symptoms of depression as the outcome. As all the studies measured depression using varying psychometric scales, the effect size measure was represented by the standardized mean differences (SMD), which compares the scores of the treatment to control group post-intervention. The effect size was calculated by subtracting the average score of the intervention group from that of the control group, and dividing the result by the pooled standard deviations. A positive effect size indicates that the intervention group had superior effects to the control group. In a clinical treatment setting, effect sizes of 0.8, 0.5 and 0.2 are considered to be large, moderate and small, respectively [28]. At a population level, when considering universal prevention interventions, smaller effect sizes are considered relevant.

If more than one measure for symptoms of depression (for example, the General Health Questionnaire (GHQ) and the Beck Depression Inventory (BDI)) was used in one study, the measure that was designed specifically for measuring depressive symptoms (that is, the BDI) was chosen for inclusion in the analysis. In the studies that included two intervention groups, SMD were computed for each treatment-control comparison, and the number of subjects in the control group was evenly divided among the intervention groups to ensure that each participant was only included once in the analysis. Adjustments were made for clustered RCTs.

A meta-analysis was performed in R v.2.15.2 statistical programming language with the metafor v.1.6 package for R [29]. For the outcome scores, the pooled mean effect sizes are expressed as SMD with 95% confidence intervals (95% CI). The studies were weighted by the inverse-variance method. As considerable heterogeneity due to population and methodological diversity was expected, we calculated pooled effect size estimates using the random effects model. The random effects model is a more conservative approach that assumes that all studies are estimating different effects resulting from variations in factors such as study population [30], sampling variation within and between studies, and as a result produces wider confidence intervals [31].

To test for heterogeneity, effect sizes were measured using Cochran’s Q-statistic, for which a *P* <0.1 was regarded as significant heterogeneity [32]. As the Cochran’s test only indicates the presence of heterogeneity and not its magnitude, we also reported the I^2^ statistic, which estimates the percentage of outcome variability that can be attributed to heterogeneity across studies. An I^2^ value of 0% denotes no observed heterogeneity, whereas, 25% is “low”, 50% is “moderate” and 75% is “high” heterogeneity [33].

We performed a separate meta-analysis on outcome scores that explicitly measured depressive symptoms or composite mental health measures to determine whether or not the measurement instrument affected the summary estimate. A separate subgroup analysis was also conducted which included only studies testing cognitive behavioral therapy-(CBT-)based interventions as these constituted the majority intervention type.

Publication bias occurs when the published studies are unrepresentative of all conducted studies due to the tendency to submit or accept manuscripts on the basis of the strength or direction of the results [34]. We examined this form of bias through a funnel plot with the SMD plotted against the SMD standard error.

## Results

### Overview of search results and included studies

**Figure 1:**
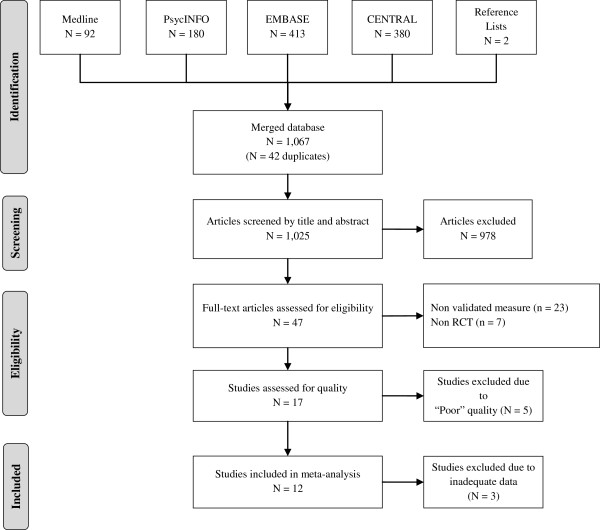
Flow diagram of study selection.

The detailed search in all databases, including CENTRAL, identified a total of 1,023 titles (following the removal of duplicates). The title and abstract of each were examined independently by two researchers (LT and MM), who identified 45 articles as relevant to the research question. Two additional articles were identified by analyzing the reference lists of the studies identified from the above strategy. None of the identified studies had utilized a clinical diagnostic tool to rule out current mental health diagnosis. Among the studies using validated self-reported measures of depression, none selected a non-depressed sample at baseline. As a result, the review was restricted to studies where diagnoses or highly symptomatic individuals were not excluded. A further independent appraisal (by LT and SH) of the full text version of these articles resulted in 17 studies meeting the criteria for quality assessment [35-50]. Figure 1 shows the flow diagram of study selection.

Two researchers (LT and MW) independently assessed the quality of the studies (N = 17). An inter-rater reliability of 0.6 (Cohen’s kappa coefficient, κ) was computed from the standard equation [51]. A consensus method was used to resolve disagreement. Following this process, 12 studies were found to be of at least a “fair” quality with final assessment scores ranging from 16 to 23 [37-40,43-45,47-50]. Five studies were excluded from the meta-analysis due to poor quality [35,36,41,42,46].

### Meta-analysis

Effect sizes (SMD) could be calculated directly using data extracted from eight of the studies [11,37,39,40,43,44,48,50]. As the two clustered RCTs [44,48] did not appear to account for the design effect in their analyses, we calculated the design effect and effective sample size based on the methods described in the Cochrane Handbook [32]. When the intra-cluster correlation (ICC) was not provided, we assumed a large ICC of 0.1.

Four authors [38,45,47,49] were contacted for missing data, out of which three [38,47,49] responded to our email requests. While two no longer had access to the data requested, we were able to obtain sufficient additional information from Ahola *et al*. [49] for effect size calculations, yielding a total of nine studies [37,39,40,43,44,48-50] for inclusion in the meta-analysis. Table 2 presents a summary of study characteristics of the included studies. Five of the studies were CBT-based [11,37,43,49,50], two were focused on mental health literacy [40,44], one was an exercise-based intervention [39] and one was based on team-based participatory intervention [48]. The interventions based on CBT principles used a variety of related techniques, including psycho-education, inoculation training, behavioral modification, stress management, and acceptance and commitment therapy. The focus of these sessions was usually on either stressful situations encountered in the workplace or more general carer management. All studies involved face-to-face interactive training and some form of health education. Each study intervention also involved multiple sessions with the exception of one study [44], which comprised a single four-hour session. Most interventions encouraged participants to undertake ‘homework’ outside of the individual sessions, with one study also providing some ongoing individual feedback via email exchanges [43].

**Table 2:** Summary of characteristics of workplace universal prevention studies included in meta-analysis

**Study**	**Subjects (sample size)**	**Design**	**Intervention description**	**Measure(s)**	**Follow-up**	**Results**
Ahola *et al.* (2012) [49]	Employees from private and public sectors (n = 718)	Individual RCT	1. Resource-enhancing group intervention ‘Towards Successful Seniority’ based on career management preparedness. 2. Wait-list control group	BDI	7 months	Significant reduction in the total symptom load of depression in the intervention group compared to the group. The intervention had no statistically significant effect on those with depression symptoms at baseline.
Atlantis *et al.* (2004) [39]	Casino employees (n = 73)	Individual RCT	1. Combined aerobic and weight training exercise with behavior modification intervention to improve mental health and quality of life outcomes. 2. Wait-list control group	DASS	24 weeks	Depression scales improved significantly for the treatment group relative to the wait-list controls.
				SF-36		
Bond and Bunce (2000) [37]	Employees (n = 90) in large media organization	Individual RCT	1. Acceptance and Commitment Therapy (ACT) aiming to enhance an individual’s ability to cope with work-related strain. 2. Innovation Promotion Program (IPP) that helped individuals identify and innovatively change causes of occupation strain. 3. Wait-list control group	GHQ-12	27 weeks	Improvements in mental health and work-related variables were found following both interventions. GHQ scores were significantly lower in the ACT condition than IPP.
				BDI		
						BDI score decreased in IPP condition from T1 to T2 and in the ACT condition from T2 to T3.
Kitchener and Jorm (2004) [40]	Employees (n = 301) in two large government departments	Individual RCT	1. Mental Health First Aid training course: to help people in mental health crises and/or in the early stages of mental health problems. 2. Wait-list control group	SF-12	5 months	Significantly greater improvement in mental health (depression and anxiety) for intervention group.
Limm *et al*. (2011) [11]	Lower and middle level managers in an international manufacturing plant (n = 174)	Individual RCT	1. Stress management intervention: using psychodynamic, conflict and emotion-focused principles and CBT. 2. Wait-list control group	HADS	12 months	Depression improvements were higher in intervention group but did not reach statistical significance.
Mino *et al*. (2006) [43]	Workers (n = 58) in the Program Development Section within a manufacturing company	Individual RCT	1. Stress management program: based on CBT approach, muscle relaxation training and counselling via email. 2. Control group: No intervention	GHQ-30	3 months	GHQ score decreased in both groups but was not significant. Significant improvement in the depressive symptoms (CES-D) was observed in the stress management group compared to the control group. In the multiple regression analysis, stress management significantly reduced depressive symptoms (CES-D).
				CES-D		
Takao *et al*. (2006) [44]	Supervisors (n = 46) of a Japanese sake brewery and their subordinates (n = 226)	Cluster RCT	1. Supervisor-based education program for employee mental health promotion and active listening training (consulting skills combined with role-playing exercises). 2. Wait-list control group	BJSQ	3 months	Intervention effects were not significant for psychological distress for both male and female subordinates. However, there were significant intervention effects for psychological distress in young male subordinates in white-collar occupations.
Tsutsumi *et al*. (2009) [48]	Workers (n = 97) in 11 assembly lines in a medium-sized manufacturing company	Cluster RCT	1. Team-based participatory intervention based on active employee involvement, shared work-related goals, and action planning to improve the workplace stress reduction. 2. Control group: No organized activities provided	GHQ	13 months	GHQ scores significantly deteriorated in control lines; scores of intervention lines remained the same.
Vuori *et al*. (2012) [50]	Workers (n = 718) across 17 participating government and private organizations	Individual RCT	1. One week resource building group intervention: career management and mental health workshop using active learning process, social modelling, gradual exposure and role playing. 2. Control group: literature package with career management related information	BDI	7 months	The program significantly decreased depressive symptoms and intentions to retire early, and increased mental resources among the intervention group compared to the controls.

BDI, Beck Depression Inventory; BJSQ, Brief Job Stress Questionnaire; CBT, cognitive behavioral therapy; CES-D, Center for Epidemiologic Studies for Depression; DASS, Depression, Anxiety, and Stress Scales; GHQ, General Health Questionnaire; HADS, Hospital Anxiety and Depression Scale; SF-12, 12-item Short Form Health Survey; SF-36, 36-item Short Form Health Survey.

### Effects of workplace intervention program compared to control conditions

**Figure 2:**
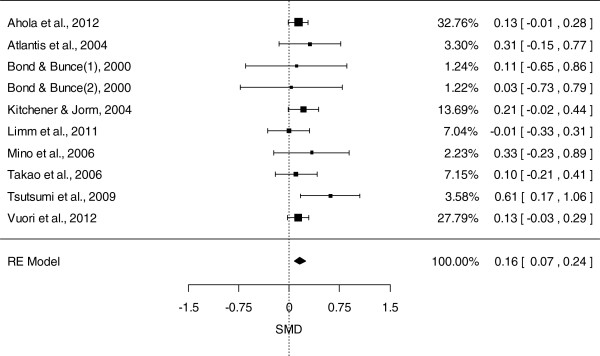
Meta-analysis examining the impact of workplace universal interventions on depression measures.

**Figure 3:**
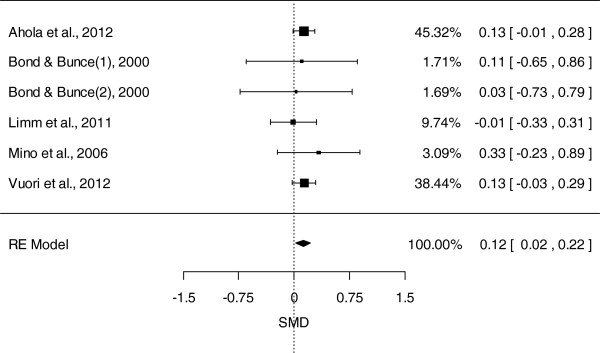
Subgroup analysis of cognitive behavioral therapy -based universal prevention interventions on depression measures.

Figure 2 presents the SMDs at post-test and the pooled mean effect size using the random effects model (REM), for the nine studies included in the meta-analysis. The overall mean difference between the intervention and control groups was 0.16 (95% CI: 0.07, 0.24, *P* = 0.0002), with effect sizes varying from small negative effects (d = -0.01) to moderate positive effects (d = 0.61). No heterogeneity was detected (Q = 6.56; I^2^ = 0%; *P* = 0.68). As noted above, more than half of the included studies (n = 5) examined the impact of interventions based on CBT. A separate meta-analysis including only CBT-based intervention studies was conducted, the results of which are presented in Figure 3. The overall mean difference between CBT-based interventions and the control groups was 0.12 (95% CI: 0.02, 0.22, *P* = 0.01), indicating a positive effect for CBT-based interventions. There was no evidence of heterogeneity in this analysis (Q = 5; I^2^ = 0%; *P* = 0.93).

Because the instruments employed to measure depressive symptoms differed widely across the studies, we conducted basic subgroup analysis examining scores from measures of composite mental health (for example, GHQ) and specific measures of depressive symptoms (for example, Center for Epidemiologic Studies for Depression (CES-D), BDI). Both types of outcome measures produced an overall positive effect, although composite measures (d = 0.23, 95% CI: 0.08, 0.39, *P* = 0.0032) produced larger differences in SMDs relative to explicit measures of depressive symptoms (d = 0.13, 95% CI: 0.04, 0.23, *P* = 0.0075).

### Sensitivity analysis

Although adjustment for effective sample size was made for the clustered RCTs [44,48], we conducted a sensitivity analysis excluding reports adopting this study design. The removal of these studies did not significantly affect the pooled effect size (d = 0.14, 95% CI: 0.06, 0.23). As one study [37] included two intervention groups, we conducted another sensitivity analysis merging the two intervention groups to create a single pair-wise comparison. The mean effect size remained unchanged (d = 0.16, 95% CI: 0.07, 0.24). Finally, we examined the five studies that were excluded from the study due to poor quality. Four of these studies did not provide sufficient information for further analysis [35,36,42,46]. We were able to obtain the means and standard deviations from only one of these studies to include in the meta-analysis [41]. However, including this study made no difference to the pooled effect size (d = 0.16, 95% CI: 0.08, 0.25). As the results of the other four studies were mostly positive, inclusion of these studies would have been unlikely to influence the pooled effect size.

### Analysis of publication bias

Due to the limited number of studies included in our analysis, it was difficult to determine the presence of asymmetry by inspection of the funnel plot. Hence, we also used Egger’s linear regression model to statistically test for funnel plot asymmetry [52]. Additionally, we computed the Rosenberg’s fail-safe number, which gives the number of unpublished studies needed to reduce the effect to non-significance [53]. The Egger’s regression test for asymmetry (*P* = 0.4262) suggested that there was no significant publication bias; the fail-safe number of 26 obtained using the Rosenberg approach indicates that 26 unpublished studies reporting no effect would be needed to reduce the pooled effect estimate to non-significance.

## Discussion

This is the first published systematic review and meta-analysis examining randomized controlled trials of universal interventions to prevent the development of depression at work. Our results indicate that a range of different depression prevention programs produce small but overall positive effects in the workplace. When analyzed separately universally delivered CBT-based interventions significantly reduced levels of depressive symptoms among workers. These results demonstrate that appropriate evidence-based interventions in the workplace should be part of efforts to prevent the development of depression.

While the effect sizes demonstrated for universal symptom reduction were relatively small, this does not mean they would not have considerable impact at a population level. Universal interventions are never likely to produce large individual effect sizes, but when translated to an entire workforce, the overall impact can be substantial. Within our review, there were some individual studies which were able to demonstrate larger effect sizes. For example, Tsutsumi *et al.* found that when a team-based participatory intervention was used to improve workplace stress reduction, there was significant deterioration of GHQ scores in the control group while the intervention group remained the same, with an overall moderate effect size of 0.6 [48]. Interestingly, this study was also the only intervention based at the organizational level, as opposed to all other studies that were based at the individual level, suggesting the benefits of organizational level approaches deserves further attention.

The main strengths of this review are the very detailed systematic search strategy, the clear defined inclusion criteria and the objective assessment of the methodological rigor of each included study. Despite these strengths, there are a number of other limitations to this review. First, due to the limited number of studies identified, we were unable to make direct comparisons to determine which type of interventions was most effective or whether an intervention based on psychosocial education is more effective over participatory-based interventions. However, there were adequate numbers of CBT-based intervention trials to perform a separate meta-analysis in order to establish the effectiveness of this particular group of interventions. Second, given that the study populations were randomized, we conducted the meta-analysis under the assumption that pre-test depression scores were the same for the control and treatment groups. The majority of studies in our meta-analysis assessed and reported that no significant differences were present in the pre-test scores; however, there were several studies that did not perform such analyses. Thus, if the pre-test scores among the treatment arms are significantly different for these studies, some bias may be introduced. Third, as self-report measures were used in all studies, our conclusions are limited to reductions in symptoms rather than clinical diagnosis. The combination of self-report symptoms together with the fact participants were not blinded to the type of intervention they received, may have introduced some bias via the Hawthorn effect. An additional problem with the measures used in many of the studies included in this review is that they combined both depression and anxiety symptoms. Our sensitivity analysis demonstrated that the beneficial effects of universal prevention remained even when only studies with pure depressive symptoms measures were included, suggesting there is a true impact on depression. Whether there is an additional and potentially even greater impact on anxiety symptoms remains unclear. Fourth, as workplace interventions are not often reported or published in academic material, there may be some publication bias in this area of research with publications only reporting significant results. However, the regression tests we conducted to examine the possibility of publication bias indicated that this was unlikely to alter our results. Finally, as we adopted a search strategy with only English publications, there is a possibility that there might be non-English universal prevention publications that were not identified.

While no studies of true prevention were identified, the finding of effective universal symptom reduction is important as it demonstrates that universally delivered programs are effective at improving employee mental health. We defined true prevention studies as needing to select a non-depressed sample at baseline and to examine the incidence at follow-up [13,20]. One of the key problems in attempting to undertake intervention studies of true prevention is the sample sizes required to gain sufficient statistical power. Cuijpers has demonstrated this with a series of calculations, which showed that in order to be able to demonstrate that a true preventative program could reduce the rates of new onset depression over one year by 15%, both the experimental and control groups would need to consist of over 30,000 participants [54]. While unable to definitively demonstrate true primary prevention, the studies of universally delivered interventions identified in this review have the advantage of accurately demonstrating the impact of interventions delivered to an entire sample of unselected workers, which is often more practically and ethically feasible in a work situation.

Prevention of mental health problems in a general community setting is still a relatively new area of research [8], although recent community-based research has provided promising results on the feasibility of prevention as a way of reducing the incidence and overall burden of depression [13]. The results of our review and meta-analysis suggest that the workplace is an alternative location in which preventative mental health programs can be successful. The workplace provides a unique location in which the majority of working-age adults can be engaged. The high cost of depression for employers, in terms of sickness absence and reduced work performance [55,56], also provides an opportunity for private organizations to be encouraged to help fund prevention programs; although further economic analysis of the costs and financial benefits of work-based universal interventions will be needed to further this case. One of the main limitations of wide-spread implementation of the types of interventions included in this review is cost, both financial and time. Most of the interventions tested required substantial amounts of face to face teaching or group training time, ranging from a single four-hour session to a year-long intervention of redesigning the work environment. There is some emerging evidence that e-health technologies may be able to assist in meeting some of these practical challenges [57]. Internet-based CBT has been shown to be effective as a treatment for depression and anxiety and is able to enhance mental well-being in a community setting [58,59]. While there are some early indications that computer-aided interventions are well received in the workplace [55], the effectiveness of universal work-based e-health prevention strategies remains unknown.

## Conclusions

In conclusion, the current review demonstrates there is good quality evidence that universal mental health interventions can reduce the overall level of depression symptoms in a workforce. Specifically, workplace CBT-based interventions are effective at universal symptom reduction for depression. More research is required to determine the extent to which such interventions can prevent new cases of depression and to establish cost effective and practical strategies for wide scale implementation. Overall, the results of this review provide support for work-based mental health interventions and add to the imperative that depression should no longer be ignored in workplace health promotion programs.

## Abbreviations

BDI: Beck Depression Inventory; BJSQ: Brief Job Stress Questionnaire; CBT: cognitive behavioral therapy; CENTRAL: Cochrane Central Register of Controlled Trials; CES-D: Center for Epidemiologic Studies for Depression; DASS: Depression, Anxiety, and Stress Scales; GHQ: General Health Questionnaire; HADS: Hospital Anxiety and Depression Scale; ICC: intra-cluster correlation; RCT: randomized controlled trial; REM: random effects model; SF-12: 12-item Short Form Health Survey; SF-36: 36-item Short Form Health Survey; SMD: standardized mean differences.

## Competing interests

The authors declare that they have no competing interests. HC is the Executive Director of the Black Dog Institute which provides mental health training for workplaces.

## Authors’ contributions

LT and SH devised the study. LT, MM and SH carried out the systematic literature search. LT, MW and SH extracted, analyzed and interpreted the data and wrote the first draft of the manuscript. All authors read and contributed to subsequent versions, and approved the final manuscript.
