# Erratum: preventing the development of depression at work: a systematic review and meta-analysis of universal interventions in the workplace

**DOI:** 10.1186/s12916-014-0212-4

**Published:** 2014-11-13

**Authors:** Leona Tan, Min-Jung Wang, Matthew Modini, Sadhbh Joyce, Arnstein Mykletun, Helen Christensen, Samuel B Harvey

**Affiliations:** University of New South Wales, School of Psychiatry, Black Dog Institute, Prince of Wales Hospital, Hospital Road, Randwick, NSW 2031 Australia; Norwegian Institute of Public Health, Bergen, Norway; Black Dog Institute, Prince of Wales Hospital, Hospital Road, Randwick, NSW 2031 Australia; St George Hospital, Gray Street, Kogarah, NSW 2217 Australia

## Erratum

## Authors’ correction note:

Upon reviewing our recently published review [1], we discovered that two of the nine studies we identified in our meta-analysis were in fact using results from the same dataset. We would not normally include the same data twice in a meta-analysis, so since discovering this we have re-run the analysis removing one of the studies [2]. This resulted in a very slight change in the pooled effect size for workplace universal interventions on depression measures (was originally 0.16, now 0.17, 95% CI: 0.07, 0.27) and the subgroup analysis of cognitive behavioural therapy–based universal prevention interventions on depression measures (remains unchanged at 0.12, but with a slight alteration in the confidence intervals, 95% CI: −0.01, 0.24). These are very minor changes in effect sizes which do not change our overall conclusions regarding the effectiveness of workplace universal interventions.

## Corrected text:

(Page 1: Abstract, final sentence of Results)

Please replace:

A separate analysis using only CBT-based interventions yielded a significant SMD of 0.12 (95% CI: 0.02, 0.22, *P* = 0.01).

With the amended text:

A separate analysis using only CBT-based interventions yielded a SMD of 0.12 (95% CI: −0.01, 0.24, P = 0.07).

(Page 5: Results. Overview of search results and included studies)

Please add the following text at the end of the second paragraph:

However, upon closer inspection two studies [2,3] were found to have used the same dataset which resulted in one [2] of these being excluded from the meta-analysis.

(Page 6: Effects of workplace intervention program compared to control conditions, first paragraph)

Please replace:

Figure 2 presents the SMDs at post-test and the pooled mean effect size using the random effects model (REM), for the nine studies included in the meta-analysis. The overall mean difference between the intervention and control groups was 0.16 (95% CI: 0.07, 0.24, P = 0.0002), with effect sizes varying from small negative effects (d = −0.01) to moderate positive effects (d = 0.61). No heterogeneity was detected (Q = 6.56; I2 = 0%; P = 0.68). As noted above, more than half of the included studies (n = 5) examined the impact of interventions based on CBT. A separate meta-analysis including only CBT-based intervention studies was conducted, the results of which are presented in Figure 3. The overall mean difference between CBT-based interventions and the control groups was 0.12 (95% CI: 0.02, 0.22, P = 0.01), indicating a positive effect for CBT-based interventions. There was no evidence of heterogeneity in this analysis (Q = 5; I2 = 0%; P = 0.93).

With the amended text:

Figure 2 presents the SMDs at post-test and the pooled mean effect size using the random effects model (REM), for the eight studies included in the meta-analysis. The overall mean difference between the intervention and control groups was 0.17 (95% CI: 0.07, 0.27, P = 0.0009), with effect sizes varying from small negative effects (d = −0.01) to moderate positive effects (d = 0.61). No heterogeneity was detected (Q =6.44; I2 = 0%; P = 0.60). As noted above, more than half of the included studies (n = 5) examined the impact of interventions based on CBT. A separate meta-analysis including only CBT-based intervention studies was conducted, the results of which are presented in Figure 3. The overall mean difference between CBT-based interventions and the control groups was 0.12 (95% CI: −0.01, 0.24, P = 0.07), indicating a small effect, of borderline statistical significance, for CBT-based interventions. There was no evidence of heterogeneity in this analysis (Q =1.28; I2 = 0%; P = 0.86).

(Page 9: Discussion, first paragraph)

Please delete:

When analyzed separately universally delivered CBT-based interventions significantly reduced levels of depressive symptoms among workers.

(Page 10: Conclusions)

Please replace:

Specifically, workplace CBT-based interventions are effective at universal symptom reduction for depression.

With amended text:

There is emerging evidence that workplace CBT-based interventions are likely to be effective at universal symptom reduction for depression.

## References

[1] Tan L, Wang MJ, Modini M, Joyce S, Mykletun A, Christensen H, Harvey SB (2014). Preventing the development of depression at work: a systematic review and meta-analysis of universal interventions in the workplace. BMC Med.

[2] Vuori J, Toppinen-Tanner S, Mutanen P (2012). Effects of resource-building group intervention on career management and mental health in work organizations: randomized controlled field trial. J Appl Psychol.

[3] Ahola K, Vuori J, Toppinen-Tanner S, Mutanen P, Honkonen T (2012). Resource-enhancing group intervention against depression at workplace: Who benefits? A randomised controlled study with a 7-month follow-up. Occup Environ Med.

